# Fluid therapy in adults having non-cardiac surgery: A narrative review

**DOI:** 10.1016/j.aicoj.2025.100006

**Published:** 2026-01-19

**Authors:** Michael Eichlseder, Romina Schweikert, Abdelkader Serir, Bernd Saugel

**Affiliations:** a Division of Anaesthesiology and Intensive Care Medicine 1, Medical University of Graz, Graz, Austria; b Department of Anesthesiology, Center of Anesthesiology and Intensive Care Medicine, University Medical Center Hamburg-Eppendorf, Hamburg, Germany; c Outcomes Research Consortium®, Houston, Texas, United States of America

**Keywords:** Anesthesia, Cardiac output, Colloid, Crystalloid, Fluid responsiveness, Goal-Directed therapy, Hemodynamic monitoring

## Abstract

**Background:**

Adequate intraoperative fluid therapy is essential, as both uncorrected fluid loss and excessive fluid administration are associated with increased complications. However, current practice varies widely. In this narrative review, we examine current concepts of intraoperative fluid therapy in adults having non-cardiac surgery, focusing on fluid type, volume of fluid, and fluid administration strategy.

**Results:**

Balanced crystalloids, compared to unbalanced crystalloids, more closely resemble the body's natural electrolyte composition. However, moderate intraoperative volumes of 0.9% saline do not seem to increase complications. Colloid fluids additionally contain larger molecules exerting colloid osmotic pressure and can be divided into synthetic and natural colloids. While concerns about synthetic colloids, especially hydroxyethyl starch, persist in intensive care medicine, intraoperative trials suggest that giving moderate volumes of hydroxyethyl starch is safe. The natural colloid human albumin theoretically offers a more favorable safety profile than synthetic colloids, but large randomized trials justifying the increased costs through improved outcomes are missing. Fluid administration strategies include calculation-based strategies, the concept of fluid responsiveness, and goal-directed fluid therapy. Calculation-based strategies rely on formulas to estimate fluid requirements. For patients having elective major non-cardiac surgery, a mildly positive intraoperative fluid balance (1–2 liters at the end of the procedure) is generally recommended. The concept of fluid responsiveness aims to assess the current hemodynamic status and evaluate whether a patient’s cardiac output increases after fluid administration. However, even if fluid responsive, fluids should only be administered if there are additional clinical or metabolic signs of hypovolemia or tissue hypoperfusion. Goal-directed fluid therapy aims to optimize hemodynamics via treatment strategies by titrating fluids, vasoactive drugs, and inotropes to predefined hemodynamic target variables. Yet, goal-directed fluid therapy did not reduce complications compared to routine care in patients having non-cardiac surgery in recent multicenter trials.

**Conclusion:**

The optimal type of fluid for intraoperative fluid therapy remains uncertain and limited volumes of unbalanced crystalloids and hydroxyethyl starch appear to be safe in surgical patients. A mildly positive intraoperative fluid balance is generally recommended for patients having major non-cardiac surgery. Fluid responsiveness can help guide fluid administration, but should not be the only factor leading to fluid administration.

## Background

All patients having major surgery are given intravenous fluids. Adequate intraoperative fluid therapy is important as there is a U-shaped relationship between the volume of fluid administered and postoperative complications [1]. Yet, fluid therapy varies among anesthesiologists – and the attending anesthesiologist appears to be the primary prognostic factor for the administered fluid volume, rather than patient-related or surgery-related factors [2,3]. This might represent uncertainty and knowledge gaps regarding fluid therapy.

In this narrative review, we therefore examine current concepts of intraoperative fluid therapy in adults having non-cardiac surgery, with a focus on the optimal fluid type, volume, and administration strategy. We compare restrictive and liberal approaches used in calculation-based concepts, critically reappraise the concept of fluid responsiveness, and debate goal-directed fluid therapy. Furthermore, we discuss future directions and open research questions regarding intraoperative fluid therapy.

## Type of fluid

Crystalloid and colloid fluids are available for intraoperative use. Crystalloid fluids contain water-soluble electrolytes in varying concentrations but lack proteins or insoluble molecules [4]. Colloid fluids additionally contain larger molecules exerting colloid osmotic pressure [5].

### Unbalanced versus balanced crystalloids

Crystalloid fluids can be unbalanced or balanced. Unbalanced crystalloids, such as 0.9% sodium chloride (commonly referred to as “normal saline”), have traditionally been the cornerstone of intravenous fluid therapy due to their isotonic nature, widespread availability, and low cost [6,7]. However, their high chloride content can lead to hyperchloremic metabolic acidosis, which itself has been associated with adverse outcomes, including acute kidney injury [8,9]. In contrast, balanced crystalloid solutions more closely resemble the body's natural electrolyte composition with a lower chloride content due to the substitution of anions (for example lactate, acetate, or malate), thereby reducing the likelihood of acid-base disturbances and hyperchloremia [4,8]. While using balanced *versus* unbalanced crystalloid fluids may improve outcomes in critically ill patients [10], a balanced crystalloid did not improve patient outcome in the only major intraoperative trial, the single-center cluster randomized SOLAR trial [11]: 8616 patients having elective orthopedic or colorectal surgery received either lactated Ringer’s solution or 0.9% sodium chloride according to departmental practice that changed every two weeks during the course of the trial. There was no meaningful difference between patients receiving lactated Ringer’s *versus* 0.9% sodium chloride in the primary composite outcome of in-hospital mortality and in-hospital major postoperative complications (5.8% *versus* 6.1%; relative risk: 1.16, 95%-confidence interval (CI): 0.89–1.52) nor in the incidence of postoperative acute kidney injury (6.6% *versus* 6.2%; relative risk: 1.18, 99.3%-CI: 0.99–1.14) [11]. Notably, the average volume of fluids patients were given during surgery was only 1.9 liters. Although the SOLAR trial suggests that giving limited volumes of 0.9% saline during non-cardiac surgery does not increase complications, multicenter randomized trials investigating the impact of perioperative fluid therapy with larger volumes of 0.9% saline are needed.

### Intraoperative use of colloids

Colloid solutions can be divided into synthetic colloids – such as hydroxyethyl starch (HES), gelatin, and dextran – and natural colloids, for example human albumin. Colloids contain large molecular structures causing osmotic pressure and thus longer remain within the intravascular space. Theoretically, this provides more efficient plasma volume expansion and requires less fluid to achieve similar hemodynamic endpoints compared to fluids without colloid osmotic effects [12,13]. However, clinical evidence from critically ill patients has raised concerns regarding the safety and efficacy of synthetic colloids [[14], [15], [16]]. These concerns include increased incidence of acute kidney injury with HES, coagulopathy and, especially with gelatin-based colloids, anaphylactic reactions [17].

The three largest intraoperative trials did not discover increased rates of complications when using HES in elective non-cardiac surgery patients – however, the crystalloid sparing effect was lower than anticipated (approximately 1:1.4) [[18], [19], [20]]. The multicenter PHOENICS trial investigated the non-inferiority in safety of 6% HES 130/0.4 (maximum dose of 30 ml/kg) compared to a balanced crystalloid solution for the treatment of hypovolemia in 1985 patients having elective abdominal surgery with an expected blood loss ≥500 ml [18]. The primary endpoint, mean change from pre- to postoperative cystatin-C-based estimated glomerular filtration rate, did not differ between the two groups (HES group: -3.4 (±17.7) ml/min/1.73 m^2^, balanced crystalloid group: -1 (±17.1) ml/min/1.73 m^2^) and non-inferiority was demonstrated (p < 0.001) [18]. The key secondary endpoint, a composite of mortality and major postoperative complications in the first 90 days after surgery, also did not differ between the two groups [18]. However, the net fluid balance from induction of anesthesia to the first postoperative morning was lower in the HES group compared to the balanced crystalloid group (600 (±2900) ml *versus* 1200 (±2700) ml, p = 0.0002) [18]. In a 3-center trial, 1102 patients having abdominal surgery were randomized to a Doppler-guided intraoperative volume replacement strategy with 6% HES 130/0.4 (maximum dose 1500 ml) or lactated Ringer’s solution [19]. Neither the primary composite outcome (cardiac, pulmonary, infectious, gastrointestinal, renal, and coagulation complications within 30 days after surgery) nor the primary safety outcome (postoperative change in serum creatinine up to 6 months after surgery) was different between the groups [19]. The primary outcome occurred in 18% of patients receiving HES and in 20% of patients receiving lactated Ringer’s solution (relative risk 0.90, 95%-CI: 0.65–1.23). There was no evidence that the colloid HES provoked acute kidney injury during hospitalization or up to 6 months after surgery (although 6-months creatinine values were available only a small fraction of patients) [19]. In the FLASH trial, conducted in 20 hospitals in France, 818 adults with increased risk of postoperative acute kidney injury having major abdominal surgery were randomly assigned to receive either 6% HES 130/0.4 diluted in 0.9% saline or pure 0.9% saline up to a maximum of 30 ml/kg [20]. If exceeding 30 ml/kg, open label fluids were administered. There was no meaningful difference in the incidence of the composite primary outcome (death or major postoperative complications) within 14 days after surgery (36% in HES group patients and 32% in saline group patients; relative risk: 1.10, 95%-CI: 0.91–1.34) [20]. The three trials thus suggest that using HES in moderate doses during the day of non-cardiac surgery does not increase complications.

The natural colloid human albumin theoretically offers a more favorable safety profile than synthetic colloids [21,22]. Human albumin may potentially improve endothelial function and reduce the risk of kidney injury without impairing coagulation [[23], [24], [25]]. However, it remains largely unknown if albumin improves outcomes compared to crystalloids or other colloidal fluids as available evidence largely stems from observational clinical or animal studies. This necessitates large-scale randomized trials to establish definitive guidance regarding albumin usage during non-cardiac surgery [21]. High cost and limited availability [6], as well as neutral results of trials in critically ill patients [26,27], might be among the reasons why a major randomized controlled trial in non-cardiac surgery has not yet been performed.

## Volume of fluid and fluid administration strategies

**Fig. 1:**
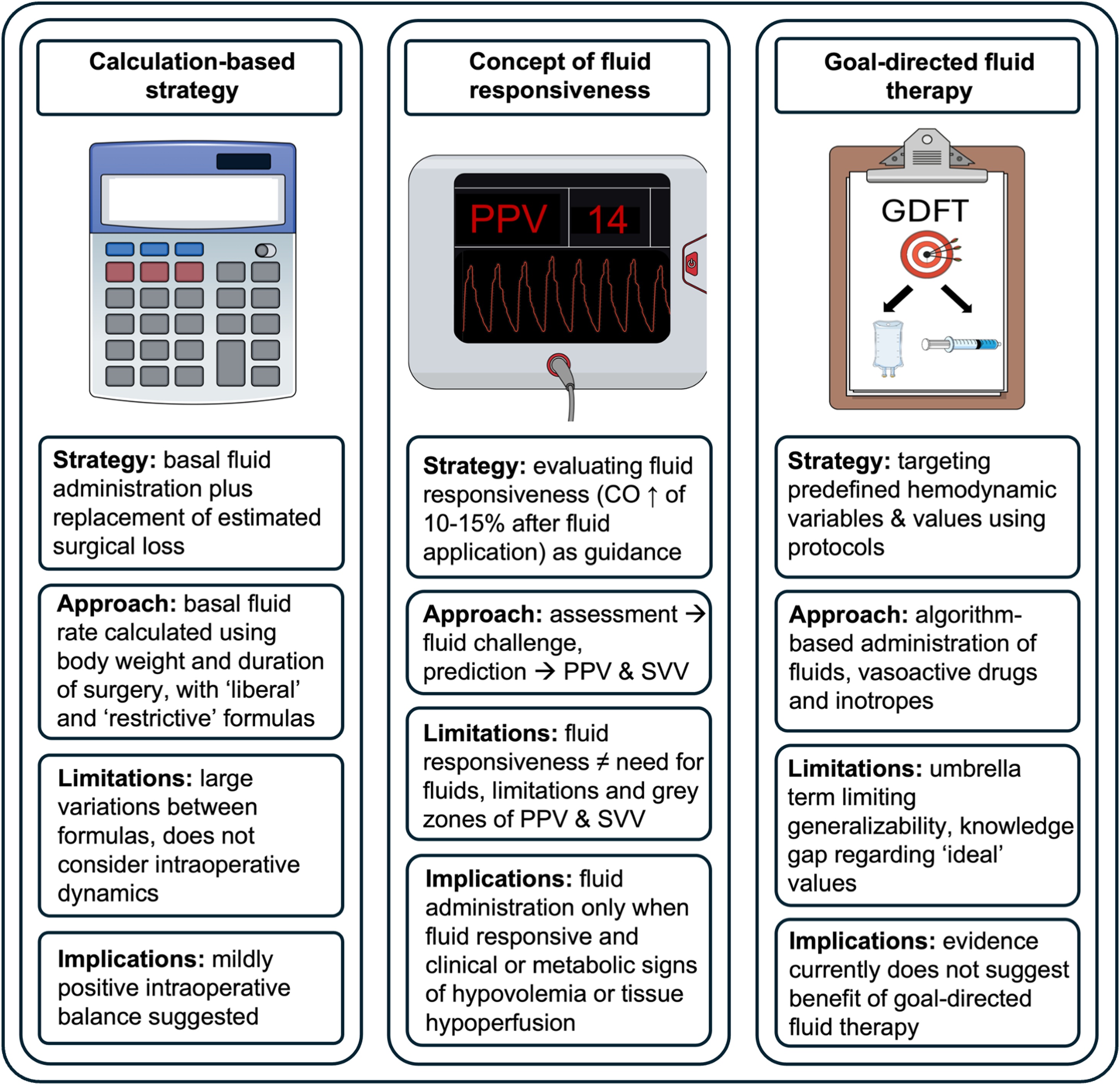
Volume of fluid and fluid administration strategies. CO = cardiac output, PPV = pulse pressure variation, SVV = stroke volume variation.

To achieve an adequate fluid balance, both uncorrected fluid loss and excessive fluid administration must be avoided. Uncorrected fluid loss can lead to decreased perfusion of vital organs with consecutive end organ damage, for example acute kidney injury [28,29]. Excessive fluid administration can cause tissue edema and is associated with higher rates of pulmonary complications, poor wound healing, ileus, and acute kidney injury [1,[30], [31], [32]]. There are several strategies for fluid administration during surgery: calculation-based strategies rely on formulas to estimate fluid requirements. The concept of fluid responsiveness aims to assess the current hemodynamic status and evaluate whether a patient’s cardiac output increases after fluid administration. Goal-directed fluid therapy uses protocols to guide hemodynamic therapy aiming at predefined hemodynamic variables. This section will provide an overview of these three fluid administration strategies (Fig. 1).

### Calculation-based strategies

**Fig. 2:**
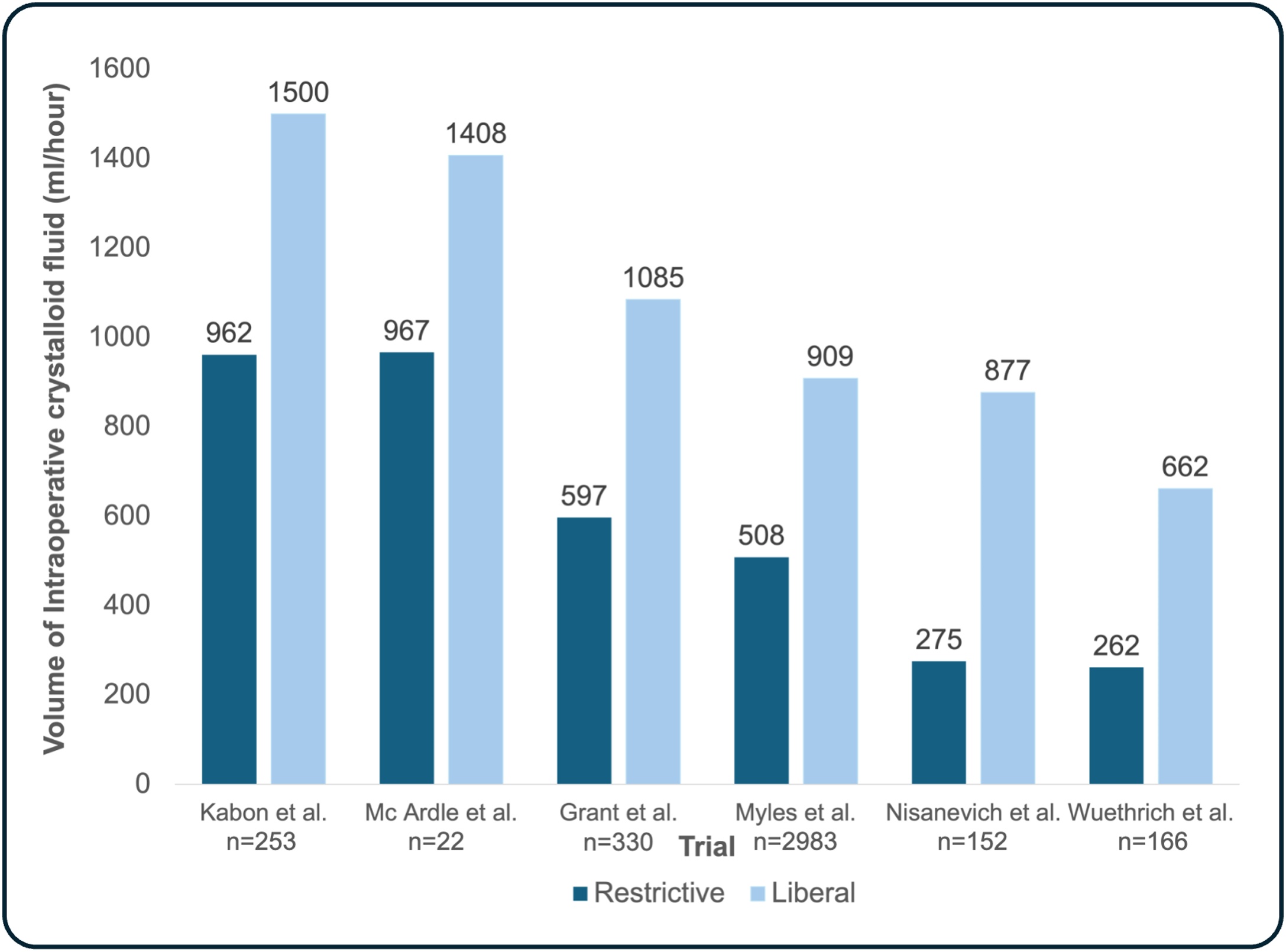
Volume of administered intraoperative crystalloid fluid per hour in trials comparing restrictive and liberal formulaic approaches. Depicted trials: Kabon et al., n = 253, mean (standard deviation) volume of total intraoperative crystalloid fluid (restrictive approach: 2500 (±1300), liberal approach: 3900 (±1900) ml) [43], McArdle et al., n = 22, mean (standard deviation) volume of total intraoperative crystalloid fluid (restrictive approach: 2626 (±478), liberal approach: 3309 (±216) ml) [40], Grant et al., n = 330, median (25% percentile, 75% percentile) volume of total intraoperative crystalloid fluid (restrictive approach: 2050 (650, 5130 ml), liberal approach: 3563 (1050, 7550) ml) [44], Myles et al., n = 2983, median (25% percentile, 75% percentile) volume of total intraoperative crystalloid fluid (restrictive approach: 1677 (1173, 2294 ml), liberal approach: 3000 (2100, 3850) ml) [29], Nisanevich et al., n = 152, median (25% percentile, 75% percentile) volume of total intraoperative crystalloid fluid (restrictive approach: 1230 (490, 7180 ml), liberal approach: 3670 (1880, 8800) ml) [39], Wuethrich et al., n = 166, median (25% percentile, 75% percentile) volume of total intraoperative crystalloid fluid (restrictive approach: 1700 (700, 4000 ml), liberal approach: 4300 (2800, 6200) ml) [42]. ml = milliliters

Historically, fluid volume was typically calculated based on the patient's body weight, along with the type and duration of the surgical procedure [33]. Using these parameters, the calculations aimed to account for the patient’s basal fluid requirement, blood loss, and fluid loss due to ‘third spacing’ [33]. ‘Third spacing’ – a concept increasingly challenged today – refers to the fluid shift from the intravascular space into a ‘third space’ [34,35]. The fluid shift into this anatomically ill-defined extracellular space was believed to be caused by capillary leakage, inflammation, and evaporation as a consequence of surgical trauma and the inflammatory response [35]. Textbooks suggested to give up to 10–15 ml/kg/h of fluid during major non-cardiac surgery to compensate for ‘third spacing’ [36,37]. This regularly resulted in up to 7 liters of fluid being applied during major non-cardiac surgery and a weight gain of 3 kilograms on the first postoperative day [38]. This so-called ‘liberal’ approach of calculated fluid administration was challenged and compared against a ‘restrictive’ approach in several smaller trials around the turn of the millennium [[38], [39], [40]]. Some of the early trials showed that restrictive – compared to liberal – fluid administration reduced complications and length of hospital stay, despite a high variation in their formulaic approach (Fig. 2) [[38], [39], [40], [41], [42]]. However, improved outcomes with the restrictive approach could not be replicated in larger trials.

In the multi-center RELIEF trial, 3000 patients having major abdominal surgery were randomized to either restrictive fluid therapy aiming at a ‘net zero balance’ (with a 5 ml/kg-fluid bolus before anesthetic induction and an intraoperative rate of 5 ml/kg/h) or liberal fluid therapy (with a 10 ml/kg-fluid bolus before anesthetic induction and an intraoperative rate of 8 ml/kg/h which could be reduced after 4 hours) [29]. In both groups, estimated blood loss was substituted with either blood or colloid fluid in a 1:1 ratio. Whereas the median (25% percentile, 75% percentile) volume of administered crystalloid fluid differed between the groups (restrictive approach: 1677 (1173, 2294 ml) *versus* liberal approach: 3000 (2100, 3850) ml), there was no significant difference in the primary outcome, disease-free survival after one year [29]. Interestingly, a higher incidence of acute kidney injury was observed in patients assigned to restrictive fluid therapy [29]. The RELIEF trial substantially influenced recent international consensus recommendations suggesting a cumulative positive fluid balance of 1–2 liters at the end of the case in patients having elective major non-cardiac surgery [45,46].

In another trial, 330 patients having pancreatic resection were randomized to either restrictive (6 ml/kg/h crystalloid during surgery, bolus of 100–250 ml in case of hypotension, blood loss substituted 1:1 with 5% albumin, and 60 ml/h crystalloid after surgery) or liberal management (12 ml/kg/h crystalloid during surgery, bolus of 100–250 ml in case of hypotension, blood loss substituted 1:1 with 5% albumin, and 125 ml/h crystalloid after surgery) [44]. Despite varying volumes of median administered crystalloid fluid on the day of surgery (2050 (650–5130) ml *versus* 3563 (1050–7550) ml), complication rates and length of hospital stay were similar between the two groups [44].

With approximately 3 liters of fluid given during major abdominal surgery, both trials followed a rather modest liberal approach and compared it to a truly restrictive strategy. Therefore, the results should not be used to support excessive infusion of fluids during surgery. Rather, both under administration, which increased acute kidney injury in the RELIEF trial [29], and over administration of fluids should be avoided in patients having major non-cardiac surgery. A mildly positive intraoperative fluid balance is recommended [45,46].

### Concept of fluid responsiveness

Fluid responsiveness is defined as a 10–15% increase in cardiac output as a result of fluid administration [47]. By either assessing or predicting fluid responsiveness, the concept aims for tailoring fluid administration to each individual patient’s current clinical condition to avoid giving fluids if an increase in cardiac output is unlikely.

Fluid responsiveness is assessed by performing a fluid challenge. A predefined volume of fluid is given during a short period of time and the patient’s hemodynamic response, ideally cardiac output, is evaluated [48]. However, each fluid challenge leads to fluid administration. Therefore, fluid challenges must not be considered a test but an intervention. This intervention has the risk of fluid unresponsive patients receiving an unnecessary and potentially harmful drug. This risk can be reduced by applying smaller volumes of fluid. ‘Mini fluid challenges’ of 100 ml (instead of 500 ml boluses which are typically used) may be sufficient to assess fluid responsiveness [49].

To avoid unnecessary fluid administration by repeatedly performing fluid challenges, numerous tests to predict fluid responsiveness have been developed. Static cardiac filling pressures, including central venous pressure, lack the ability to predict fluid responsiveness [50,51]. Central venous pressure, despite being valuable in assessing right heart function, is therefore not recommended to predict fluid responsiveness during surgery [46,52]. Methods for predicting fluid responsiveness include the passive leg raise test [53], dynamic preload variables (pulse pressure variation and stroke volume variation) [51,54], ultrasound-guided vena cava assessment [55], end-expiratory occlusion tests [56], tidal volume challenges [57], and the sigh maneuver [58]. Since most of the tests have been developed in the intensive care setting, several of them have limitations during surgery. Dynamic preload variables are applicable during surgery and have been investigated thoroughly in this setting.

**Fig. 3:**
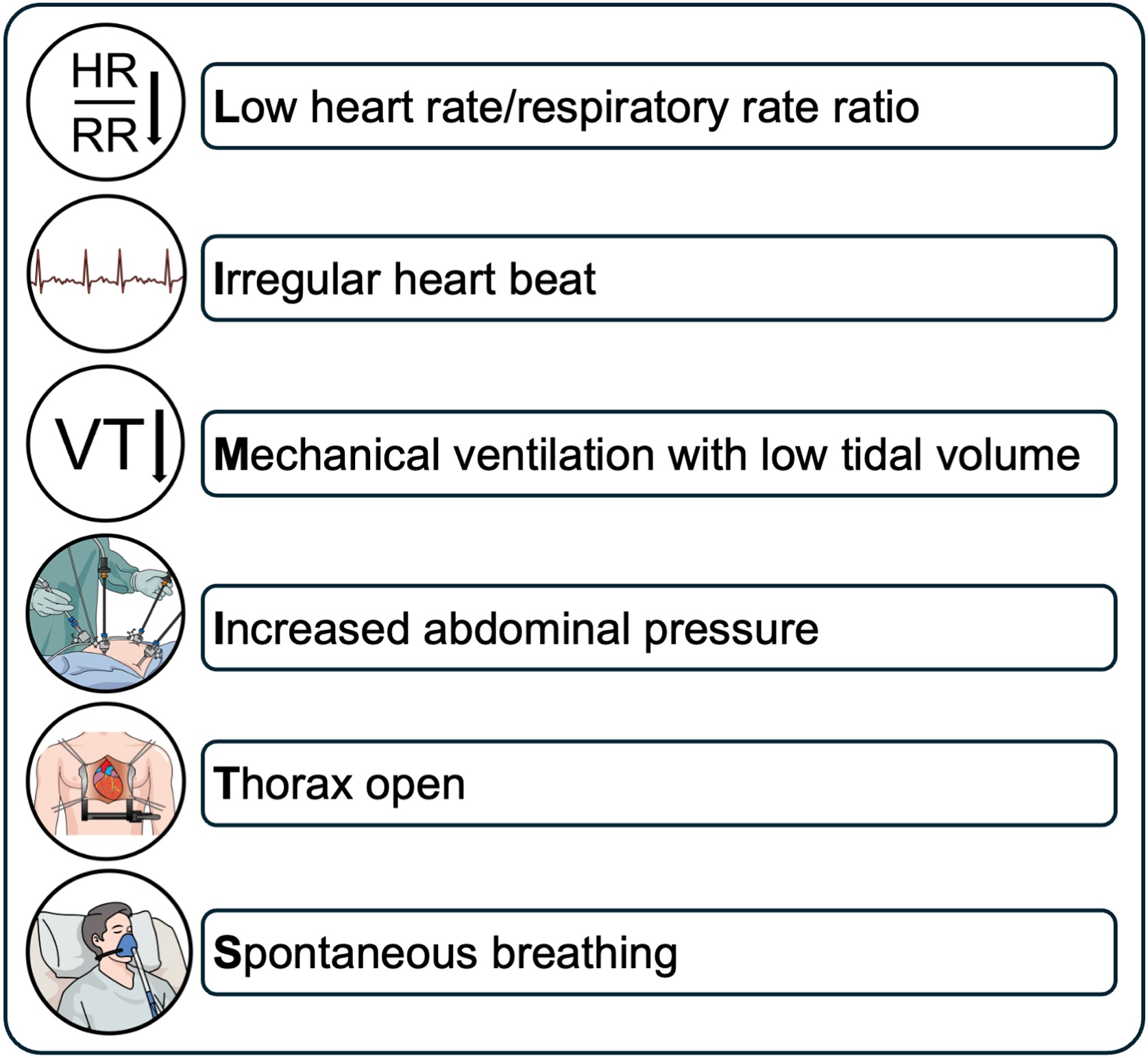
Conditions limiting the ability of dynamic preload variables to predict fluid responsiveness. Adapted from Michard et al. [63].

Dynamic preload variables include pulse pressure variation (PPV) and stroke volume variation (SVV). PPV can be calculated using variables derived from a regular invasive blood pressure monitor as only pulse pressure (PP) is required (PPV (%) = $\frac{PPmax-PPmin}{PPmean}*100)$ [51]. SVV requires additional technical equipment for stroke volume (SV) calculation (SVV (%) = $\frac{SVmax-SVmin}{SVmean}*100)$ [54]. Both variables are based on heart-lung interactions and work on the physiological principle that mechanical ventilation induces cyclic variations in ventricular loading, with higher values in fluid responsive patients. In detail, in the inspiratory phase during mechanical ventilation, the right ventricular preload decreases due to reduced venous return from increased intrathoracic pressure while right ventricular afterload increases through elevated transpulmonary pressure. Therefore, right ventricular stroke volume is lowest at end-insufflation. The subsequent reduction in right ventricular stroke volume delays left ventricular filling, reducing left ventricular stroke volume after a transit time of approximately two to four heart beats. Large left ventricular stroke volume variations suggest biventricular preload responsiveness, whereas stability indicates preload unresponsiveness in at least one of the ventricles [59]. A threshold of 11% has been proposed for both PPV and SVV to be predictive of fluid responsiveness [60]. However, a grey zone, where predictive ability is limited, has been reported for PPV values between 9% and 13% [61]. This likely also applies to SVV. Additionally, to provide adequate accuracy, sinus rhythm and mechanical ventilation with a tidal volume of at least 7−8 ml/kg are necessary [62]. Low heart rate/respiratory rate ratio, cardiac arrhythmias, mechanical ventilation with low tidal volumes, high intraabdominal pressure, open thorax situations and spontaneous breathing reduce the ability to predict fluid responsiveness drastically (Fig. 3) [63]. Keeping both the grey zone and the above-mentioned limitations in mind, PPV and SVV can help predict whether a patient is fluid responsive or not.

Importantly, fluid responsiveness is a physiological state and simply indicates that the heart can increase its left ventricular stroke volume when more blood returns to the heart and thus increases end-diastolic volume. Fluid responsiveness is also a physiological state in patients with intravascular normo- or even hypervolemia and a healthy heart, as the Frank Starling mechanism enables matching of right and left ventricular cardiac output, adapting cardiac output to changes in preload due to exercise and posture, and compensating for transient changes in heart rate. Consequently, the presence of ‘fluid responsiveness’ does not mean that a patient needs fluids and should therefore not automatically lead to fluid administration [46,52]. Fluids should only be administered if there are additional clinical or metabolic signs of hypovolemia or tissue hypoperfusion [46,52].

### Goal-directed fluid therapy

Intraoperative goal-directed fluid therapy is an umbrella term [64] describing intraoperative treatment strategies aiming to maintain adequate oxygen delivery to end-organs by titrating fluids, vasoactive drugs, and inotropes to predefined hemodynamic target variables [65]. Meta-analyses suggest that goal-directed fluid therapy may reduce postoperative morbidity and mortality [66,67]. However, among the more than 100 trials on goal-directed fluid therapy, most are small single-center trials [68] that may overestimate true treatment effects due to potential publication bias and lower methodological quality [[69], [70], [71]]. Additionally, changes in clinical practice over time (including the increased use of minimally invasive surgery) and improvements in perioperative care might also explain why benefits reported in previous smaller trials could not be confirmed in recent large trials which failed to show a reduction in complications.

A multicenter trial including 482 patients having high-risk abdominal surgery in the Netherlands found neutral results when comparing cardiac output-guided goal-directed fluid and inotrope therapy against routine care during the first 24 h of the perioperative period [72]. In the intervention group, fluid responsiveness was predicted primarily using SVV (≥12%) when the cardiac index was below the target. If patients were predicted as being fluid responsive, a 500 ml bolus of either crystalloid or colloid was administered. If predicted fluid unresponsive, inotropic support was initiated or increased. The primary outcome, average number of major complications per patient within 30 days after surgery, did not meaningfully differ (0.79 *versus* 0.69; p = 0.19) [72].

In the multicenter iPEGASUS trial, 318 patients having open abdominal surgery were randomized to cardiac index-guided fluid and inotrope therapy or to routine care [73]. The primary outcome, the incidence of a composite outcome of moderate and major complications within 28 days after surgery, was not reduced by cardiac index-guided fluid therapy. Just contrary to the hypothesis, the incidence of the primary outcome occurred in 55% of the patients assigned to cardiac index-guided fluid therapy and in 46% of the patients assigned to routine care (p = 0.038) [73].

The OPTIMISE II trial included 2498 patients having major surgery of the gastrointestinal tract in 55 hospitals in 11 countries [74]. Patients were randomized to cardiac output-guided fluid therapy with low dose inotrope infusion or routine management without cardiac output monitoring. There was no difference in the primary endpoint, postoperative infection in the first 30 days after surgery (23.2% *versus* 22.7%; adjusted odds ratio: 1.03, 95%-CI: 0.84–1.25). However, patients assigned to cardiac output-guided fluid therapy with low dose inotrope infusion had more acute cardiac events within 24 h after surgery (3.0% *versus* 1.7%; adjusted odds ratio: 1.82, 95%-CI: 1.06–3.13). Acute cardiac events did not differ 30 days after surgery [74].

Recent randomized trials therefore do not suggest that goal-directed fluid therapy reduces complications compared to routine care in unselected patients having non-cardiac surgery. One possible explanation for this might be that goal-directed fluid therapy algorithms generally follow the assumption that cardiac output – that is mainly determined by metabolic demand – should be maximized. However, this might not represent the metabolic needs of an anesthetized patient. In addition, substantially different treatment strategies and target values emerged under the term of goal-directed fluid therapy, limiting its generalizability [64,68]. This variability in target values also indicates a knowledge gap regarding ‘ideal’ values. Furthermore, the attempt to subsume fluid therapy and hemodynamic monitoring into one algorithm might be a too complex task, as already stated by Shoemaker in the 1970s after proposing protocols for hemodynamic optimization [75]. Additionally, routine care, serving as the comparator, has likely improved over the years.

## Open research questions

**Fig. 4:**
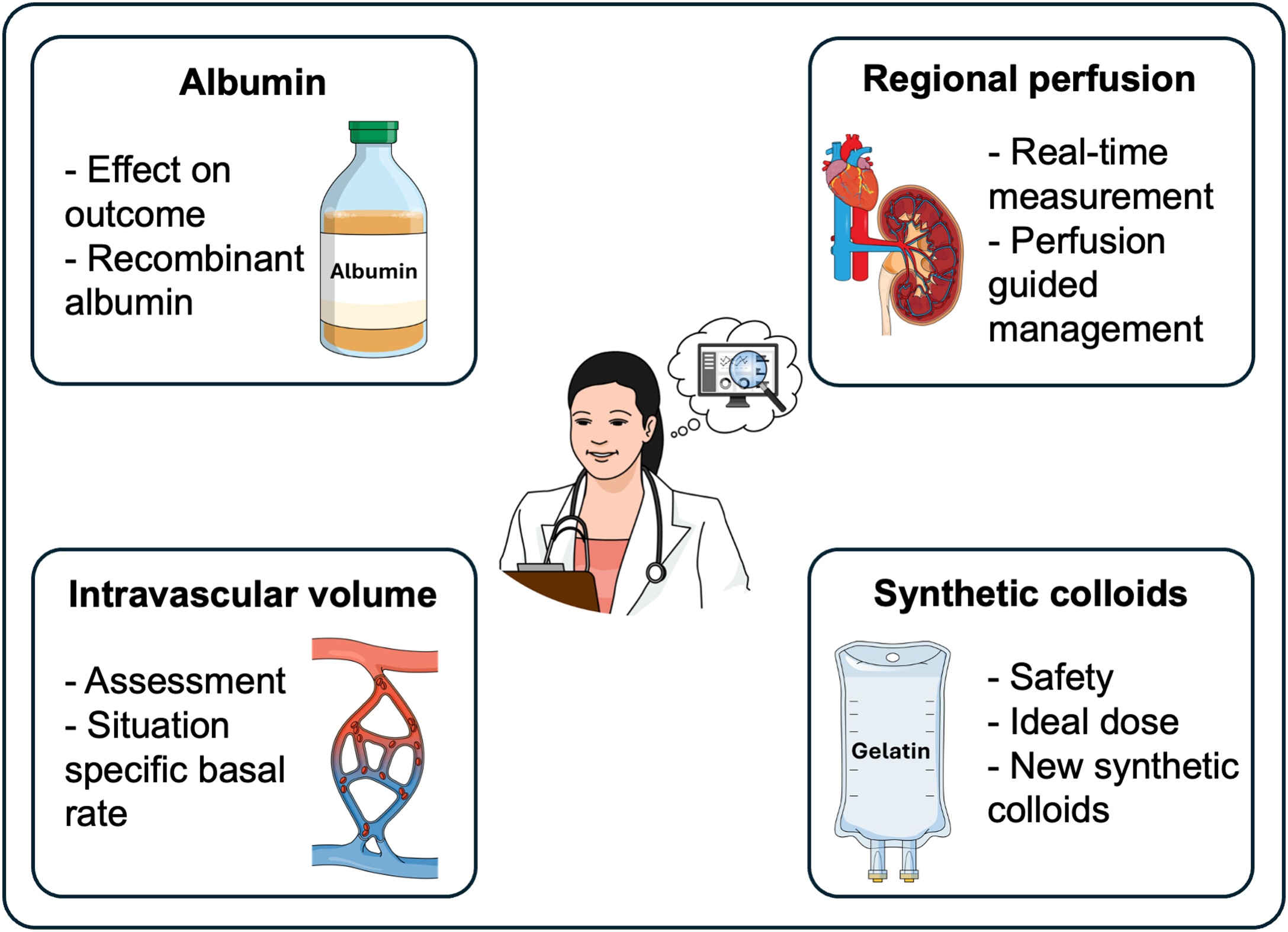
Open research questions regarding intraoperative fluid therapy.

To advance perioperative care, critical unanswered questions regarding intraoperative fluid therapy must be addressed in future research (Fig. 4). Using colloids to increase oncotic pressure in selected patients appears physiologically reasonable. However, previous research on colloids mainly focused on HES. While small volumes of HES may be safe during elective non-cardiac surgery, it remains uncertain whether this is also true for other synthetic colloids. This is especially important in patients with contraindications to HES, such as patients with impaired renal function. Trials are needed to determine whether other synthetic colloids, such as gelatin-based solutions, or the natural colloid albumin can be safely administered and can improve outcomes in certain populations of surgical patients. In addition to clinical research on existing fluids, novel pharmacological approaches may provide new therapeutic possibilities. For example, recombinant albumin, so far only studied in healthy volunteers [76], could potentially be an alternative to human albumin (which is limited and expensive).

Beyond selecting the ‘ideal’ type of fluid, the optimal timing and strategy for fluid administration also requires further investigation. Baseline patient factors, surgery, and general and neuraxial anesthesia can each influence the intravascular fluid status in different ways. The wide variability in patient physiology and perioperative conditions makes it challenging to establishing fluid administration strategies that are broadly applicable across surgical populations. Basal fluid administration to cover baseline needs, supplemented by fluid boluses to compensate for surgery-related losses, appears physiologically reasonable. However, optimal basal infusion rates for different types of surgery have yet to be determined. Goal-directed fluid therapy has not demonstrated a reduction in complications compared to routine care in multicenter randomized trials (Table 1). To enhance targeted hemodynamic management, future efforts should focus on identifying reliable methods to assess intravascular volume status alongside real-time measurements of regional tissue perfusion.

**Table 1:** Summary of the discussed trials.

Author/trial name	Year	Design	Population	Intervention	Comparison	Primary Outcome	Results	Additional findings
SOLAR [11]	2020	Cluster randomized trial (single center)	Orthopedic or colorectal surgery (n = 8616)	Lactated Ringer (intraoperative fluid)	0.9% sodium chloride (intraoperative fluid)	Composite outcome (mortality and major postoperative renal, respiratory, infectious, or hemorrhagic complications) during the hospital stay	Neutral result	Neutral result regarding postoperative acute kidney injury
PHOENICS [18]	2025	Randomized controlled trial (53 centers)	Major abdominal surgery (n = 1985)	6% HES 130/0.4 for the treatment of hypovolemia due to blood loss	Balanced crystalloids for the treatment of hypovolemia due to blood loss	Mean difference in change from pre- to postoperative cystatin-C-based estimated glomerular filtration rate	Non-inferiority of HES compared to balanced crystalloids	Lower net fluid balance from induction of anesthesia until the first postoperative morning in the HES group
Kabon et al. [19]	2019	Randomized controlled trial (3 centers)	Major abdominal surgery (n = 1102)	6% HES 130/0.4 (intraoperative volume replacement, maximum volume 1.5 liters, Doppler-guided)	0.9% sodium chloride (intraoperative volume replacement, Doppler-guided)	Composite outcome (cardiac, pulmonary, infectious, gastrointestinal, renal, and coagulation complications) within 30 days after surgery	Neutral result	Neutral results regarding postoperative serum creatinine 6 months after surgery
FLASH [20]	2020	Randomized controlled trial (20 centers)	Major abdominal surgery (n = 818)	Diluted 6% HES 130/0.4 (intraoperative volume replacement, maximum HES volume 30 ml/kg)	0.9% sodium chloride (intraoperative volume replacement)	Composite outcome (death or major postoperative complications) within 14 days after surgery	Neutral result	–
RELIEF [29]	2018	Randomized controlled trial (47 centers)	Major abdominal surgery (n = 3000)	Restrictive (5 ml/kg bolus at induction +5 ml/kg/h) calculation-based management	Liberal (10 ml/kg bolus at induction +8 ml/kg/h) calculation-based management	Disease-free survival after one year	Neutral result	Higher incidence of acute kidney injury in the restrictive group
Grant et al. [44]	2016	Randomized controlled trial (single center)	Pancreatic resection surgery (n = 330)	Restrictive (6 ml/kg/h during surgery) calculation based management	Liberal (12 ml/kg/h during surgery) calculation based management	Clavien-Dindo grade III complications within 60 days after surgery	Neutral result	Neutral result regarding length of hospital stay
De Waal et al. [72]	2021	Randomized controlled trial (5 centers)	Major abdominal surgery (n = 482)	Cardiac output-guided goal directed fluid and inotrope therapy	Standard of care	Composite outcome (postoperative complications) in the first 30 days after surgery	Neutral result	–
iPEGASUS [73]	2024	Randomized controlled trial (5 centers)	Open major abdominal surgery (n = 318)	Cardiac index-guided goal directed fluid and inotrope therapy to maintain optimized postinduction cardiac index	Standard of care	Incidence of moderate and major complications within 28 days	Higher incidence in the cardiac index-guided group	–
OPTIMISE II [74]	2024	Randomized controlled trial (55 centers)	Major gastrointestinal surgery (n = 2498)	Cardiac output-guided fluid therapy with low dose inotrope infusion	Standard of care (no cardiac output monitoring)	Postoperative infections within 30 days after randomization	Neutral result	Higher incidence of acute cardiac events in the cardiac output guided group

HES = hydroxyethyl starch.

In the future, closed-loop algorithms and decision-support systems may reduce variability in intraoperative fluid administration and improve the consistency and quality of patient care by enabling (semi)automated fluid administration based on real-time physiologic data [77,78].

## Conclusion

Adequate intraoperative fluid therapy is important – and challenging. Both uncorrected fluid loss and excessive fluid administration must be avoided to maintain tissue perfusion and prevent tissue edema. The optimal type of fluid for intraoperative fluid therapy remains uncertain. Future trials need to determine whether intraoperative fluid therapy should be primarily based on unbalanced crystalloids, balanced crystalloids, synthetic colloids, or human albumin. Unlike in critically ill patients, limited volumes of unbalanced crystalloids and HES appear to be safe in surgical patients. There are several strategies for fluid administration during surgery. Calculation-based strategies rely on formulas to estimate fluid requirements. For patients having elective major non-cardiac surgery, a mildly positive intraoperative fluid balance (1–2 liters at the end of the procedure) is generally recommended. The concept of fluid responsiveness aims to assess the current hemodynamic status and evaluate whether a patient’s cardiac output increases after fluid administration. However, the presence of ‘fluid responsiveness’ does not mean that a patient needs fluids and should therefore not automatically lead to fluid administration. Fluids should only be administered if there are additional clinical or metabolic signs of hypovolemia or tissue hypoperfusion. Intraoperative goal-directed fluid therapy uses protocols to guide intraoperative fluid administration based on predefined hemodynamic targets. In recent multicenter randomized trials, goal-directed fluid therapy did not reduce complications compared to routine care in patients having non-cardiac surgery. To advance perioperative care, future research must address critical unanswered questions related to intraoperative fluid therapy, including the optimal fluid type, volume, and administration strategy.

## CRediT authorship contribution statement

Idea and concept for the article: **BS**; Drafted the work: **ME**, **RS**, **AS**; Critically revised the work: **BS**; Figures: **ME**. All authors read and approved the final manuscript.

## Consent for publication

Not applicable.

## Ethics approval and consent to participate

Not applicable.

## Funding

No funding to declare.

## Availability of data and material

Data sharing is not applicable to this article as no datasets were generated or analyzed during the current study.

## Declaration of competing interest

**ME**, **RS** and **AS** declare that they have no competing interests.

**BS** is a consultant for and has received institutional restricted research grants and honoraria for giving lectures from Edwards Lifesciences (Irvine, CA, USA). BS is a consultant for Philips North America (Cambridge, MA, USA) and has received honoraria for giving lectures from Philips Medizin Systeme Böblingen (Böblingen, Germany). BS has received institutional restricted research grants and honoraria for giving lectures from Baxter (Deerfield, IL, USA). BS is a consultant for and has received institutional restricted research grants and honoraria for giving lectures from GE Healthcare (Chicago, IL, USA). BS has received institutional restricted research grants and honoraria for giving lectures from CNSystems Medizintechnik (Graz, Austria). BS is a consultant for Maquet Critical Care (Solna, Sweden). BS has received honoraria for giving lectures from Getinge (Gothenburg, Sweden). BS is a consultant for and has received institutional restricted research grants and honoraria for giving lectures from Pulsion Medical Systems (Feldkirchen, Germany). BS is a consultant for and has received institutional restricted research grants and honoraria for giving lectures from Vygon (Aachen, Germany). BS is a consultant for and has received institutional restricted research grants from Retia Medical (Valhalla, NY, USA). BS is a consultant for and has received honoraria for giving lectures from Masimo (Neuchâtel, Switzerland). BS is a consultant for Dynocardia (Cambridge, MA, USA). BS has received institutional restricted research grants from Osypka Medical (Berlin, Germany). BS received honoraria for giving lectures from Ratiopharm (Ulm, Germany). BS was a consultant for and has received institutional restricted research grants from Tensys Medical (San Diego, CA, USA). BS is an Editor of the British Journal of Anaesthesia.
